# Cellular senescence in cancer: molecular mechanisms and therapeutic targets

**DOI:** 10.1002/mco2.542

**Published:** 2024-04-24

**Authors:** Ping Jin, Xirui Duan, Lei Li, Ping Zhou, Cheng‐Gang Zou, Ke Xie

**Affiliations:** ^1^ State Key Laboratory for Conservation and Utilization of Bio‐Resources in Yunnan, School of Life Sciences Yunnan University Kunming Yunnan China; ^2^ Department of Oncology School of Medicine Sichuan Academy of Medical Sciences and Sichuan Provincial People's Hospital University of Electronic Science and Technology of China Chengdu Sichuan China; ^3^ Department of Anorectal Surgery Hospital of Chengdu University of Traditional Chinese Medicine and Chengdu University of Traditional Chinese Medicine Chengdu China

**Keywords:** immunosenescence, senescence, senescence‐associated secretory phenotype (SASP), senotherapeutics, tumor

## Abstract

Aging exhibits several hallmarks in common with cancer, such as cellular senescence, dysbiosis, inflammation, genomic instability, and epigenetic changes. In recent decades, research into the role of cellular senescence on tumor progression has received widespread attention. While how senescence limits the course of cancer is well established, senescence has also been found to promote certain malignant phenotypes. The tumor‐promoting effect of senescence is mainly elicited by a senescence‐associated secretory phenotype, which facilitates the interaction of senescent tumor cells with their surroundings. Targeting senescent cells therefore offers a promising technique for cancer therapy. Drugs that pharmacologically restore the normal function of senescent cells or eliminate them would assist in reestablishing homeostasis of cell signaling. Here, we describe cell senescence, its occurrence, phenotype, and impact on tumor biology. A “one‐two‐punch” therapeutic strategy in which cancer cell senescence is first induced, followed by the use of senotherapeutics for eliminating the senescent cells is introduced. The advances in the application of senotherapeutics for targeting senescent cells to assist cancer treatment are outlined, with an emphasis on drug categories, and the strategies for their screening, design, and efficient targeting. This work will foster a thorough comprehension and encourage additional research within this field.

## Graphical abstract

Senescent cells exert a double‐edged sword effect on tumor progression. Amplification beneficial effects while inhibition related protumorigenic pathways of senescence will help improve antitumor efficacy.

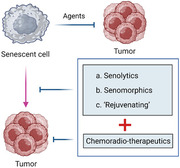

## 1 INTRODUCTION

Cellular senescence, genomic instability, telomere attrition, epigenetic changes, declining proteostasis, deregulated nutrient sensing, mitochondrial dysfunction, stem cell exhaustion, and altered intercellular communication are some of the previously reported hallmarks of aging.^1^ More recently recognized ones include disabled macroautophagy, gut dysbiosis, and chronic inflammation.^2^ The characteristics of cancer in the first generation include sustaining proliferative signaling, evading growth suppressors, resisting cell death, enabling replicative immortality, inducing angiogenesis, and activating invasion and metastasis.^3^ In the next generation, genome instability and mutation, tumor‐promoting inflammation, and evasion of immune destruction^4^ take center stage. In the emergent generation, nonmutational epigenetic reprogramming occurs, unlocking phenotypic plasticity, and uncovering the polymorphic microbiomes of senescent cells.^5^ Cellular senescence is characterized by a dynamic, multistep process of irreversible cell cycle arrest, resulting in impaired ability to repair damage, altered metabolic activity, dramatic changes in gene expression, and the gradual development of a senescence‐associated secretory phenotype (SASP) that is linked to cancer progression.^6^, ^7^, ^8^, ^9^

Senescence has long been known as a barrier to tumorigenesis due to the cycle arrest state. Oncogene‐induced senescence (OIS) resulting from continuously activated oncogenes, especially members of the RAS and BRAF families, can trigger a stable proliferation arrest that blocks unleashed promitotic activity and unscheduled DNA replication, highlighting the direct protective role of senescence against tumor initiation and development. Significantly, the tumor‐suppressive activities of senescent cells can be further enhanced by cell‐extrinsic mechanisms through the induction of senescence in adjacent cells via both the SASP and cell–cell interactions. In addition, some SASP factors can also enhance immune surveillance, which in turn accounts for the clearance of senescent tumor cells. For example, p53 cooperates with the nuclear factor kappa‐B (NF‐κB) pathway to regulate SASP factors, which activate macrophages to form a tumor‐suppressive microenvironment in a mouse model of liver cancer.^10^ Hence, the induction of tumor cell senescence has been considered an effective mechanism through which antitumor therapeutics can exert activity. This therapy‐induced senescence (TIS) can frequently be observed in patients exposed to radiation and/or chemotherapeutics.^11^, ^12^ Many anticancer drugs including alkylating agents (cisplatin, cyclophosphamide, and temozolomide), topoisomerase inhibitors (doxorubicin, etoposide, and camptothecin), and microtubule inhibitors (paclitaxel) have been identified as senescence inducers both in preclinical models and clinical trials. Radiation is used for cancer treatment owing to its ability to generate an acute burst of DNA damage, the major inducer of senescence.^13^ Collectively, the senescence induced by most anticancer treatment modalities is an integral effector that combats tumor growth not only by cell‐cycle arrest but also through immune‐mediated, cell‐extrinsic mechanisms.

**FIGURE 1:**
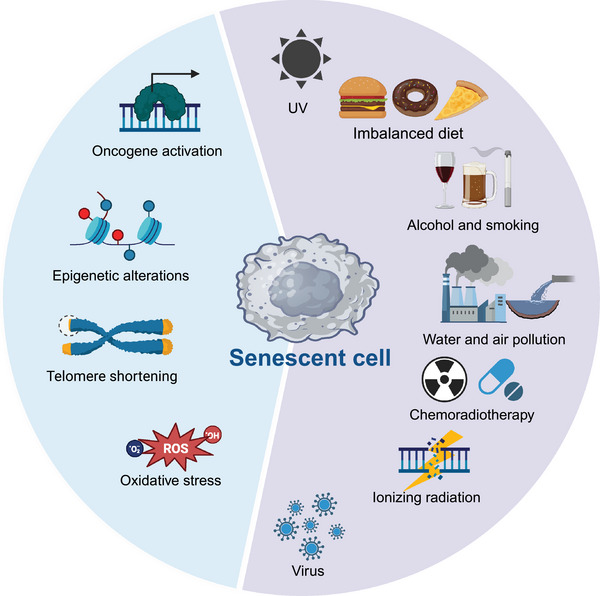
Diverse stimuli can induce cellular senescence. A plethora of endogenous or exogenous stimuli can produce a SASP. SASP, senescence‐associated secretory phenotype.

However, certain types of senescent cells exhibit aberrant behavior, which could ultimately have detrimental effects that hasten tumor development (Figure 1). It has been demonstrated that the concurrent events of cellular senescence and its multiple activities are implicated in carcinogenesis.^14^ Senescent cells displaying a SASP phenotype release inflammatory cytokines, growth factors, and chemokines that change the local tissue environment and elicit chronic inflammation.^15^ Moreover, senescence of the immune system (immunosenescence), which increases with aging and is associated with altered lymphoid organ morphology and diminished immune cell function, is also intimately linked to the emergence of malignant tumors.^16^, ^17^ Indeed, senescence has been reported to be linked to nearly every tumor trait, including initiation, development, treatment resistance, dormancy, and stemness.^18^, ^19^, ^20^, ^21^ Therefore, senescence has drawn much attention and modeling by cancer researchers, particularly after it was proposed as one of the emerging hallmarks of cancer in 2022.^5^ It is probable that a deeper investigation into the association between senescence and cancer will offer fresh perspectives on preventing the occurrence and progression of malignancies, as well as providing novel targets for tumor treatment.^22^

For the purpose of enhancing the effectiveness of antitumor treatments, efforts have been made to investigate prospective drugs and strategies to target senescent cells. Senescence induction could be a positive therapeutic approach when cellular senescence has a tumor‐suppressive role or when these senescent cells can be effectively removed.^23^ In the alternative case, inhibition or elimination of senescent cells could offer potential options for cancer treatment when senescent cells exacerbate malignant characteristics.^24^ Typically, there are two ways to combat senescence. One method is to use antisenescence drugs known as senolytics to selectively remove senescent cells,^25^ while the other strategy involves blocking induction of the senescence phenotype (SASP) by drugs called senotherapeutics.^26^ The recently reported rejuvenation approach hold promise of a third choice for targeting senescent cells. With increasing research, more and more SASP‐centered approaches are being trialed as a complement or an alternative to current senolytic treatments in targeting tumors. Moreover, the development of senotherapeutics that combine transdisciplinary technologies from several disciplines, such as biology, chemistry, nanotechnology, and immunology, is rapidly gaining interest.^27^, ^28^ To date, senotherapeutics in combination with conventional anticancer drugs has been found to afford a more successful approach to cancer therapy.^29^, ^30^ This strategy could significantly reduce the risk of cancer recurrence after treatment while also significantly lowering the side effects of cancer therapy.

Herein, we provide a thorough overview of the entire cell senescence process, including its causes, biological implications, role in tumors, and prospective senotherapeutic strategies. We also discuss the advantages and disadvantages of using targeted senescence for cancer therapy in clinical settings. Senescence modulation is still in its infancy, and it is believed that continuing research will result in substantial improvements, affording a wealth of new approaches and novel strategies for tumor therapy and prevention.

## 2 DIVERSE STRESSORS INDUCE CELLULAR SENESCENCE

Senescence occurs over time when cells experience a variety of endogenous and exogenous stresses.^31^, ^32^ Endogenous factors include oxidative stress, oncogene activation, and epigenetic changes.^33^, ^34^, ^35^ Exogenous stressors include radiation, genotoxic stress, chemotherapy, and viral infections (Figure 1).^36^, ^37^ Several classical stressors and how they elicit cellular senescence are detailed below.

### 2.1 Oncogene‐induced senescence

OIS, a crucial defense system against oncogenic events, is triggered by the DNA damage response (DDR) pathway that results from hyperproliferative oncogene expression and associated altered DNA replication.^38^, ^39^ In this process, the activation of an oncogene induces cell cycle arrest in the absence of additional mutations to ensure the removal of early neoplastic cells from the proliferative pool and the prevention of tumor cells transformation due to persistent cellular outgrowth.^34^ The first observation of OIS was a permanent G1 arrest accompanied by accumulation of p53 and p16 caused by a high level of oncogenic RAS in human cells.^40^ Since then, these findings have gradually been extended to in vitro and in vivo studies investigating OIS and tumorigenesis.^41^, ^42^, ^43^ In addition, BRAF V600E was reported to induce a similar proliferation arrest and senescence in primary human melanocytes via micRNA‐mediated suppression of AURKB.^44^ While overall these results indicated that OIS acts as a vital brake to slow carcinogenesis,^45^ the inflammation induced by OIS can sometimes promote tumor progression.^46^, ^47^

### 2.2 Exogenous stimuli

Senescent cells can accumulate as a result of external factors such as radiation therapy and chemotherapy, according to a growing body of research. In the tumor scenario, this TIS can induce chronic inflammation and cause tumor resistance to therapy, relapse, and even metastasis or paradoxically promote antitumor immunity that enhances therapeutic efficacy.^48^, ^49^, ^50^ In particular, genotoxic treatment modalities (genotoxic stress) will disrupt genomic integrity and cause DNA damage.^51^, ^52^, ^53^ Under this circumstance, cellular failsafe mechanisms such as apoptosis or senescence will be activated to stop the aberrant division of these damaged and potentially harmful cells if the DNA repair machinery fails to fix the damaged site during a transient cell cycle arrest or if intense genotoxic stress overwhelms the repair capacity.^54^ One of the best‐studied cellular stressors, heat stress, was found to induce early S‐phase cells to enter a type of cell cycle arrest that could induce cellular senescence.^55^, ^56^ Microbes can also induce senescence. For instance, the intratumoral bacterium *Stenotrophomonas maltophilia*, a common multidrug‐resistant opportunistic pathogen, was found to activate hepatic stellate cells and induce the SASP by activating the TLR4/NF‐κB/NLRP3 pathway, thereby driving the progression of liver cirrhosis toward hepatocellular carcinoma.^57^ Overall, senescence can be induced by a diverse group of stressors with multiple roles, thus offering potential targets for fighting cancer.

### 2.3 Others

These stressors generally induce a DDR or cause telomeric erosion can also eventually results in cellular senescence.^58^ DNA is susceptible to a variety of damages even under normal conditions, and the damage is usually rapidly repaired by the DDR apparatus. Senescence is frequently caused by an accumulation of DNA damage, particularly DNA double‐strand breaks (DSBs) recognized by the DDR pathway.^59^, ^60^ Through a sequence of chromatin modification processes, including the activation of ataxia telangiectasia mutated (ATM), phosphorylation of histones H2A.X and related proteins such as MDC1 and 53BP1, the accumulation of repair signals renders DNA repair sites cytologically visible as nuclear foci.^61^, ^62^ These foci act as checkpoints, stopping the cell cycle until the damage has been repaired or the damaged cell is liquidated. However, if the DNA damage persists, it will result in prolonged DDR signaling and a protracted proliferative arrest in the form of cellular senescence. Critically short telomeres are often associated with cellular senescence since telomeres are thought to be a potential determinant of life span.^63^, ^64^, ^65^, ^66^ Nondividing cells that are senescing or that have been subjected to exogenous genotoxic therapies also exhibit chronic DDR activation.^67^ For example, the E3 ubiquitin ligase, FBW7, binds to telomere protection protein 1, TPP1, in response to radiation, oxidative stress, or bleomycin, which boosts multisite poly‐ubiquitination and speeds up its degradation, leading to telomere uncapping and DDR activation.^36^

## 3 MORPHOLOGICAL FEATURES OF SENESCENCE AND THEIR ROLE IN TUMOR BIOLOGY; MOLECULAR MARKERS OF AGE‐RELATED PATHOLOGY

**FIGURE 2:**
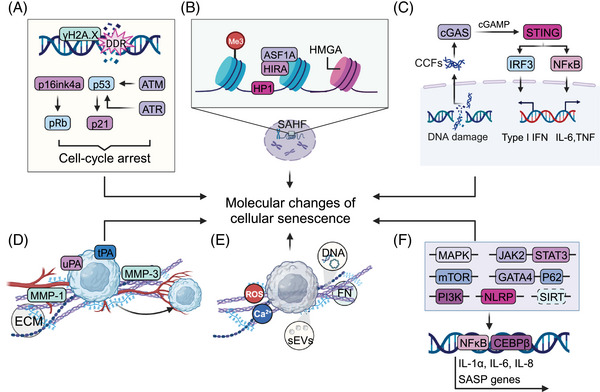
Molecular features of senescent cells. Senescent cells undergo distinct chromatin changes including the formation of (A) γH2A.X, (B) senescence‐associated heterochromatin foci, and (C) the cytoplasmic chromatin fragments (CCFs). (D) Proteases such as MMPs, serine proteases, and controllers of plasminogen activation pathway are also secreted by senescent cells. (E) Senescent cells can also synthesize sEVs to remodel the TME. (F) Multiple upstream signals have been shown to regulate senescence features, and the majority of them eventually converge to activate CCAAT/enhancer‐binding protein β (C/EBP‐β) and nuclear factor kappa‐B (NF‐κB) signaling. HMGA, high mobility group protein A; HP1, heterochromatin protein 1; SAHF, senescence‐associated heterochromatin foci; CCFs, cytoplasmic chromatin fragments; ECM, extracellular matrix; uPA, serine proteases urokinase; tPA, tissue‐type plasminogen activators; MMPs, matrix metalloproteinases; ROS, reactive oxygen species; FN, fibronectin; sEVs, small extracellular vesicles.

Senescence‐associated features typically include stable cell cycle arrest, prolonged DDR activation, senescence‐associated heterochromatin foci (SAHF), cellular enlargement, dysfunctional cellular components accompanied by unfolded protein responses (UPRs), metabolic changes such as increased senescence‐associated β‐galactosidase (SA‐β‐gal) activity and SASP^68^, ^69^, ^70^, ^71^ (Figure 2). These senescence phenotypes not only provide functional markers to characterize senescent cells but also act as an intrinsic molecular basis of age‐related pathologies such as tumors.^26^, ^67^ In‐depth explorations of the underlying molecular basis of the senescent state can offer potential targets for modulating cellular senescence as an adjunct to antitumor therapy.

### 3.1 Senescence‐associated nuclear and chromatin changes

Nuclear and chromatin undergo a complex array of molecular and morphological alterations during the senescence process. These changes include high expression of cell cycle inhibitory factors, altered nuclear morphology size and architecture, increase in chromatin condensation, histone modifications, and altered epigenetic patterns, which collectively affect gene expression and contribute to the development of age‐related diseases.

Cell cycle regulators such as cyclin‐dependent kinases (CDKs) and their inhibitors play a crucial role in regulating the cell cycle. Two important tumor suppressor pathways, p16^ink4a^‐pRb and p53‐p21, are engaged to initiate and maintain persistent cell cycle arrest in senescence.^68^, ^72^, ^73^ These four crucial factors can induce senescence by prolonged overexpression of any one of them, and they essentially sustain the senescent state by causing extensive alterations in gene expression through p53, which is an important transcription factor, and Rb, which participates in regulating the expression of a variety of genes.^74^ The Rb family is a primary target of cyclin–CDK complexes, and it functions by sequestering E2F complexes and thus inhibiting the expression of E2F target genes.^75^, ^76^ In senescence, the elevation of p16 causes Rb to remain activated, thus guaranteeing irreversible cell cycle arrest. In contrast to the relatively slow accumulation of p16, telomere damage, oxidative stress, or oncogenic stress can quickly activate p53/p21^WAF1/CIP1^.^77^ Chronic activation of p53 induced by persistent DDR signaling can also induce cellular senescence.^78^ It is noteworthy that p21 can enforce G1 arrest through binding to the cyclin E‐CDK2 and cyclin A‐CDK2 complexes to ensure that Rb is kept hypo‐phosphorylated and active in senescent cells.^79^ Senescent cells often have high expression of at least one of these important cell cycle inhibitors, even though the various senescence‐related stimuli engage different signaling pathways.^80^ This cell cycle arrest primarily suppresses the replication of cancer cells to restrain the production of a macroscopic tumor or contradictorily contribute to aggressive tumor biology and poor clinical outcome, such as dormancy, stemness, or drug resistance.^81^, ^82^

The phosphorylation of H2A.X, commonly known as γH2A.X, is widely detected in cells undergoing diverse modes of senescence. It is an important sign of DDR activation and necessary for the formation of repair complexes at DSBs.^83^, ^84^, ^85^ Members of the phosphatidylinositol 3‐kinase‐related kinase family (PI3KK), such as ATM–Chk2 and ATR–Chk1 kinases, frequently phosphorylate H2A.X, but may not be the only ones to do so.^86^ ATM is generally activated by the binding of the MRE11–RAD50–NBS1 complex to DNA breaks.^87^ The subsequent attraction of DNA damage checkpoint protein 1 (MDC1) by γH2A.X generates a complex feedback loop to maintain γH2A.X amplification and stability^88^, ^89^; therefore, a framework is developed for further recruitment and accumulation of DNA repair components, and DSB repair can therefore be carried out through nonhomologous end joining (NHEJ) and homologous recombination. Otherwise, ATR is mostly recruited to single‐stranded DNA regions at stopped replication forks in cells suffering from oxidative or replication stress.^90^, ^91^ A recent study suggested that the activation of ATM and ATR is not that simple, however, as DSBs can activate ATM and replication stress can activate ATR. The fact that H2A.X itself may be a target for other kinases and that ATM and ATR kinases phosphorylate many more target proteins than H2A.X, is one of the most obvious pieces of evidence.^92^ Intriguingly, nucleotide excision repair appears to activate ATR and produce small quantities of H2A.X, particularly when prolonged single‐stranded regions are exposed.^93^ Caffeine‐induced ATR inhibition had the opposite effect and resulted in increased S‐phase H2A.X following UV damage while having no impact on the number of focus‐positive cells, or foci per cell. Contrarily, the ATM inhibitor KU55933 and a JNK inhibitor both block S‐phase H2A.X after UV damage,^94^ indicating that the kinases most likely in charge of H2A.X production after UV insult during S phase do not include ATR, but rather ATM and JNK1.

In addition to prolonged DDR activation and permanent cell cycle arrest, chromatin organization and profound changes in the epigenome are also recognized as key features of senescence.^81^ One important aspect of these modifications is termed SAHF, the spatially ordered heterochromatic domains that can be identified as compressed foci of DNA by 4′, 6‐diamidino‐2‐phenylindole.^95^ SAHF are characterized by apparently condensed chromatin on one chromosome and reassembled with repressive markers of chromatin such as trimethylated histone H3 Lys9 and several additional proteins, including heterochromatin protein 1, histone cochaperones HIRA and ASF1A, and high mobility group protein A.^96^ Therefore, SAHF were originally proposed to transcriptionally suppress the expression of cell cycle‐promoting genes. Another chromatin feature is the existence of cytoplasmic chromatin fragments (CCFs) derived from nuclear chromatin fragments via a blebbing process.^97^ The formation of CCFs is linked to the dysfunction of nuclear structural proteins like the breakdown of the nuclear lamina that are caused by unfolding of constitutive heterochromatin domains induced by different modes of senescence stimulation.^98^ Similar to several other types of cytoplasmic DNA, such as mitochondrial DNA derived from stressed mitochondria and cDNA produced by hyperactivated long‐interspersed element‐1; CCFs affect inflammatory responses and the SASP by modulating cyclic GMP–AMP synthase (cGAS) and the adaptor stimulator of interferon genes (STING) pathway.^99^ In this context, it has been reported that low doses of histone deacetylase (HDAC) inhibitors can suppress CCFs and the SASP in senescent cells.^100^ Altogether, SAHF and CCFs and their associated regulators provide robust markers for detecting cellular senescence.

From the extensive research on senescence and its interaction with other physiological and pathological processes, several other senescent features and associated biomarkers have also been identified. It is worth mentioning that senescent cells are mostly polyploid.^101^, ^102^ This is thought to be the result of mitotic dysfunction, where cells fail to complete a full mitosis because of DNA damage, leading to the development of large cells with numerous micronuclei and decondensed chromatin.

### 3.2 Senescent cells exhibit structural and functional flaws

**FIGURE 3:**
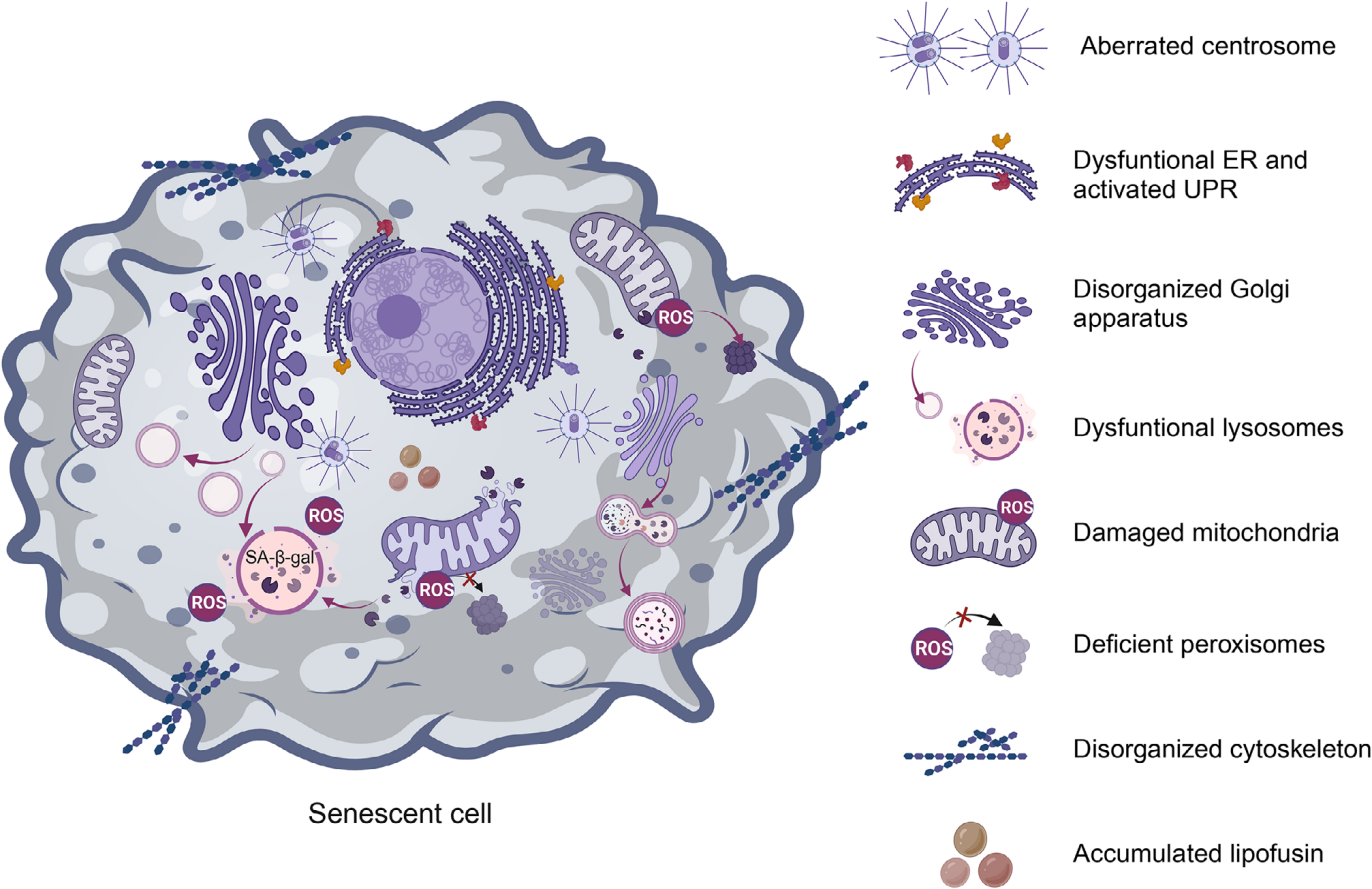
Senescent cells exhibit structural and functional flaws. Senescent cells have an enlarged nucleus, a disorganized cytoplasm, dysfunctional organelles and deficiencies in the cytomembrane. These intracellular changes could be caused by increased production of organelles with aberrant function. ER, endoplasmic reticulum; UPR, unfolded protein response.

Another characteristic of senescent cells is aberrant intracellular signaling accompanied by intracellular changes in morphology, function, and organelle mass^103^ (Figure 3). Although the exact mechanism underlying these intracellular changes in morphology and function is not fully understood, these changes could be caused by the conflict between increasing organelle production and dysfunctional organelle function, either directly or indirectly; proteins and aggregates are less effectively eliminated when organelle dysfunction exists,^104^ although senescent cells can constantly produce organelles to compensate for faulty organelle function. As senescence progresses, the newly created organelles might exacerbate the damage via elevated degradation stress and oxidative damage.^105^, ^106^

Lysosomes, the main catabolic organelles, contain a wide variety of acid hydrolases that break down nucleic acids and proteins, and senescent cells have shown pronounced changes such as increased lysosomal mass due to upregulated lysosome biogenesis.^107^, ^108^ SA‐β‐gal, the original and currently most commonly used selective biomarker for identifying cells with senescent characteristics, consistently exhibits enhanced activity.^109^ Additionally, an accumulation of lysosomes holding indigestible cargo, such as lipofuscin, sometimes known as residual or dense bodies, has been linked to an increase of lysosomal mass.^110^ Lipofuscin was found to regulate cellular senescence by increasing the expression of Bcl‐2 and conferring resistance to apoptosis.^111^ Granular buildup in the lysosomes results in an enlarged hypertrophic cellular morphology.^112^ Moreover, senescent lysosomes exhibit a reduced capacity to sense and respond to stimuli due to alterations in lysosomal pH and the activity of most lysosomal enzymes.^113^ Defective lysosomes exhibit compromised autophagic degradation of substrates as well as endocytic cargo, and this deficiency in the lysosomal/autophagy machinery might disrupt inflammasome formation.^107^ Most interestingly, the clearance of CCFs extruded from the nucleus of senescent cells also failed.^114^ This deficiency in lysosomal and autophagic degradation systems has been clearly implicated in the development of tumors.

While mitochondrial dysfunction can elicit cellular senescence, mitochondria can undergo increases in mass and size with reduced function during senescence.^106^, ^115^ Compared with lysosomal alterations, changes in mitochondria are one of the most marked senescence‐related characteristics, and they are mainly attributed to a cellular compensatory mechanism triggered by a decline in mitochondrial function.^116^ Dysfunctional mitochondria produce elevated amounts of reactive oxygen species (ROS), causing damage to DNA, lipids, and proteins, which in turn leads to an imbalance in mitochondrial dynamics and induces dramatic increases in mitochondrial volume with highly interconnected networks, aberrant morphology, and dysfunction.^117^ This, combined with the vulnerability of mitochondria to damage, remarkably disseminates oxidative stress in cells, and thus induces the senescent phenotype.^118^ In addition, signals from mitochondrial damage influence retrograde signaling pathways to the nucleus. This retrograde signal can influence nuclear transcriptional reprogramming and affects several cellular processes including proliferation, senescence, and programmed cell death.^100^, ^119^ Alternatively, severely oxidized nucleotide bases induce SSBs and DSBs and are thus involved in nuclear aspects of the senescent phenotype.^120^ Interestingly, mitochondria and lysosomes are thought to be interconnected as complex units and to play an important role in the maintenance of functional integrity and cellular homeostasis.^121^ Besides the decrease in autophagic turnover of mitochondria, defective autophagic processes can cause lipid oxidation and lipofuscin accumulation, which results in compromised formation of autolysosomes and accumulation of lysosomes loaded with indigestible lipofuscin, ultimately resulting in a lack of elimination of dysfunctional mitochondria.^113^, ^122^ Taken together, mitochondria can interact with lysosomes to demonstrate inseparable crosstalk for regulating cell senescence. In the treatment of age‐associated diseases, this crosstalk represents a fresh therapeutic frontier. Diseases associated with mitochondrial damage like cancer usually exhibit well‐known cellular compensatory mechanisms for mitochondrial expansion and increased oxidative capacity.^123^

The Golgi complex is a membrane‐bound organelle made up of a series of flattened, stacked pouches located next to the endoplasmic reticulum (ER) and near the cell nucleus.^124^, ^125^ There are two different types of polarized Golgi apparatus. The trans‐Golgi network (TGN) facilitates cargo export while the cis‐Golgi network receives cargo from the ER for processing.^126^ Therefore, the Golgi network provides not only a biosynthetic and processing hub, but also acts as an intracellular transport and secretory station for proteins and lipids.^127^ It has been reported to exhibit a large and expanded morphology and disorganized structure with altered function in senescent cells.^127^ Nonsenescent cells showed a tiny, compact Golgi apparatus in the perinuclear area, whereas senescent cells had a broad and extended structure across the cytoplasm. It is important to note that lysosomal lipid buildup from oxidized LDL has been linked to expansion of the TGN and the Golgi complex,^128^ both of which have been demonstrated to regulate tumor traits.^129^, ^130^ These findings highlight the need for additional research into the relationship between changes in the structure and function of the Golgi complex as cells senesce, and how this affects the development of tumors.

The ER, another network of membranous organelles, sometimes specifically with ribosomes attached (rough ER), participates in posttranslational modification of proteins and lipids, as well as in regulating calcium storage and secretory pathways.^131^ The UPR is activated in response to ER stress to stop the buildup of unfolded or misfolded proteins and restore ER equilibrium.^132^ Notably, when the stress exceeds a threshold and the ER becomes excessively overloaded, the UPR elicits apoptotic programmed cell death pathways.^133^ In addition to cooperating with the Golgi complex to facilitate protein processing and sorting, the ER interacts with mitochondria and lysosomes to participate in maintaining functional mitochondria and lysosomes.^134^ This coordinated regulation of autophagy, proteasome‐dependent degradation, and chaperone‐mediated protein folding preserves the integrity of the cellular proteome.^135^ According to previous observations, ER stress dysregulation and UPR activation both occur during cellular senescence and are crucial to understanding tumor biology.^136^

The centrosome is a key microtubule organizing center that helps different cell types produce bipolar mitotic spindles and provides a matrix of primary cilia. The creation of bipolar spindles and chromosomal segregation are guaranteed by the correct duplication, maturation, and separation of centrosomes.^137^, ^138^ Centrosome dysfunction (CD) not only prevents the proper transmission of genetic information but also leads to chromosomal instability, which may result in aneuploidy.^139^, ^140^ Centrosome aberrations are universal among human cancers and contribute to the senescence process.^141^ It can be either numerically aberrant or structurally abnormal. The former involves duplication cycle disruption, centrosome overduplication, mitotic abnormality, entosis, development of invadopodia, and establishment of an additional centrosome‐associated secretory phenotype.^142^ The latter results in cell movement and spreading by altering the structure of chromosomes, the localization of core proteins and their binding.^143^

Apart from the well‐established senescence‐engaged organelles described above, dysfunction of peroxisomes and the cytoskeleton is also strongly associated with senescence.^144^, ^145^ Peroxisomes are dynamic organelles enclosing a large number of enzymes involved in lipid metabolism and ROS signaling.^146^, ^147^ Peroxisome‐derived ROS interact with mitochondria to orchestrate homeostatic cellular ROS levels and function as second messengers to modulate various cellular signaling pathways. Peroxisome deficiency has been reported to cause oxidative stress and contribute to the early phases of cellular senescence.^148^ Consistent with this, it has been suggested that restoring the oxidative equilibrium in peroxisomes will slow down cellular senescence. It should come as no surprise that senescent cells show changes in the cytoskeleton and the cellular functions it affects, such as shape, cell division, motility, and intracellular trafficking. For example, vimentin is a cytoskeletal protein known to be overproduced and exist as densely bundled filaments in various types of senescent cells, where it significantly alters morphology.^149^ Additionally, nuclear blebbing, generation of micronuclei, and RhoA and Sun1/2‐regulated F‐actin cytoskeletal rigidity was discovered to be involved in progerin‐induced cellular senescence.^150^ Silencing Sun2 expression can reduce RhoA activation, cytoskeletal stiffness, and cellular senescence, revealing novel mechanical roles and correlations between cytoskeletal morphology and cellular senescence.^150^ Recently, it was discovered that kindlin‐2, a protein that belongs to the actin cytoskeleton organizing and integrin activator family, regulates cellular senescence by interacting with p53 and altering the expression of *SERPINB2* and *CDKN1A* (p21).^151^ These discoveries open up a new therapeutic window for modulating cellular senescence in the treatment of tumors.^151^

### 3.3 Secretory phenotype

Apart from the features described above, senescent cells remain metabolically active and develop a hypersecretory phenotype, SASP, another key hallmark of senescence that allows them to communicate with and affect neighboring cells.^152^ SASP components can be broadly categorized into three groups: extracellular proteases, secreted insoluble protein/nonprotein components, and soluble signaling molecules (interleukins [ILs], chemokines, and growth factors).^153^, ^154^ In this section, the major components of the SASP, the upstream mechanisms that modulate their formation and secretion, and their role in the tumor microenvironment (TME) are presented.

#### 3.3.1 Soluble signaling factors

ILs, inflammatory cytokines, and growth factors are examples of soluble signaling elements that can influence neighboring cells. IL‐6, IL‐1, and IL‐8 are among the upregulated proinflammatory cytokines in the SASP. IL‐6 is the most prominent and appears to be associated with oncogenic stress‐induced DNA damage and directly regulated by ATM–CHK2 signaling, independent of the p53 pathway.^155^ Through the interaction of IL‐6 with its receptors, gp80 and gp130, expressed on the surface of nearby cells, senescent cells can directly affect various functions.^156^, ^157^ Another IL signaling pathway that senescent cells have been shown to upregulate involves IL‐1. Senescent endothelial cells, fibroblasts, and chemotherapy‐induced senescent epithelial cells all overexpress and produce IL‐1α and IL‐1β. Similar to IL‐6, the IL‐1 receptor/Toll‐like receptor (TLR) families can influence nearby cells. The majority of senescent cells also overexpress IL‐8 (CXCL‐8), GRO‐1, and GRO‐2 (CXCL‐1 and ‐2; KC for murine CXCL‐1). These ILs are also significantly overexpressed in tumors, which has clear implications for the connection between senescence and tumorigenesis.^158^ For instance, senescent astrocytic CRT cells (a human glioma cell line) showed upregulated levels of IL6 when incubated with d‐galactose to counteract the cytotoxicity of temozolomide in U373‐MG cells. Therefore, d‐galactose treatment could lead to elevated numbers of IL‐6‐secreting senescent astrocytes, which could contribute to brain inflammation and tumor progression.^159^

Another soluble signaling factor of the SASP that plays a significant role in senescence and influences how it affects the microenvironment is the insulin‐like growth factor (IGF), which is one of the ligands in the IGF receptor network. The IGF‐binding protein (IGFBP) family, consisting of IGFBP 1−7 and their regulators IGFBP‐rP1 and IGFBP‐rP2 (also known as connective tissue growth factor), is aberrantly expressed and plays decisive roles in controlling cell senescence in a variety of cell types.^54^, ^160^, ^161^, ^162^, ^163^ IGF signaling also influences proliferation, survival, and migration of epithelial cells, and its overexpression seems to play a role in the etiology of many cancers.^164^, ^165^, ^166^ In a recent observational analysis of 20 prospective studies with Mendelian randomization, IGF‐I was positively associated with higher risk of prostate cancer, with possibly earlier onset of disease of an overall more aggressive type.^167^ Notably, IGFBP2 was shown to exert IGF‐independent extracellular and intracellular actions resulting in cell growth arrest,^168^ in addition to being highly expressed on a variety of tumor cells and influencing mitogenic IGF functions in the intercellular space.^169^ Colony‐stimulating factors (CSFs) such as GM‐CSF and G‐CSF, osteoprotegerin, prostaglandin E2 (PGE2), and the enzyme that produces it, Cox‐2, are other soluble components identified with the SASP. They have been found to be highly abundant in senescent fibroblasts and to be essential for senescence, which makes them suspects in the process of malignant transformation.^170^, ^171^

#### 3.3.2 Extracellular proteases

Proteases such as matrix metalloproteinases (MMPs), serine proteases, and controllers of the plasminogen activation pathway are also secreted by senescent cells. The MMP family members, collagenase‐1 (MMP‐1) and stromelysin‐1 (MMP‐3), show consistent overexpression in senescent cells.^172^, ^173^ Through secretion of these MMPs, senescent cells can modify the TME by shedding membrane‐associated proteins, rendering them soluble, or degrading signaling molecules and the ECM. These MMPs can cleave IL‐8, MCP‐1, ‐2, and ‐4,^174^ and others, such as MMP‐2 and ‐7, can also cleave the additional CXCL/CCL family members that make up the SASP.^175^ Senescent stromal cells may increase the permeability of nearby capillaries by the action of MMPs, exposing cancer cells to higher concentrations of mitogens, cytokines, and other secreted products. This exposure may accelerate the growth of preneoplastic cells and subsequently boost the proliferation of cancer cells. Distinct types of proteases identified in the SASP include serine proteases and plasminogen activation pathway regulators, which are both associated with the development of cancer.^176^, ^177^ This family consists of the serine proteases, urokinase, and tissue‐type plasminogen activators (uPA and tPA, respectively), the uPA receptor (uPAR), and inhibitors of these serine proteases (PAI‐1 and ‐2).^178^ It has been discovered that senescent endothelial cells, lung fibroblasts, and skin fibroblasts have elevated activity or protein level of plasminogen, which may be connected either directly or indirectly to tumor progression.

#### 3.3.3 Insoluble extracellular molecules and emerging secretory components

Senescent cells have a wide‐ranging impact on the TME by secreting insoluble molecules such as fibronectin (FN) and nonproteins as well as the aforementioned soluble cytokines and kinases.^179^ Increases in the protein level of FN, a ubiquitous glycoprotein of the extracellular matrix that binds to ECM molecules, cell‐surface receptors, and components of the cytoskeleton to affect cell shape and movement,^180^ have been observed in cellular senescence.^181^, ^182^ Numerous studies have proposed an important role of FN in the pathobiology of cancer. Targeting FN alone for cancer management, however, carries a risk because of its apparently contradictory involvement in carcinogenesis.^183^ Further research is needed to clarify whether FN engagement in the senescence process is one of the factors in the conflicting roles of FN in cancer and TMEs. The majority of nonprotein extracellular species consist of ROS, ions such as Ca^2+^,^174^ and DNA fragments released from mitochondria and nuclei. It has also been found that senescent cells release nitric oxide and ROS when the inducible nitric oxide synthase, endothelial nitric oxide synthase, and superoxide dismutase are activated. These reactive molecules are well‐established players in both cellular physiology and pathology and are known to enhance cancer cell aggressiveness.

In certain situations, senescent cells can also synthesize small extracellular vesicles (sEVs) to remodel the TME in addition to the recognized SASP components,^184^ and these sEVs are unquestionably engaged in the regulation of tumor phenotypes.^185^ For example, extracellular vesicles produced by SIRT1‐deficient senescent stromal cells can upregulate the amount of ATP binding cassette subfamily B member 4, which could modify the gene expression profile of prostate cancer cells resulting in a drug‐resistant phenotype.^186^ Further study on the association between senescence and tumorigenesis is expected to reveal more SASP components, and the investigation of these regulatory factors will provide new opportunities for treating tumors by modulating cellular senescence.

#### 3.3.4 Features of SASP and its upstream regulatory mechanisms

Most senescent cells have a core group of SASP factors, but the heterogeneity of the SASP in different cell types is closely associated with the type of senescence inducer and the duration of exposure. The levels of many secreted factors, especially the inflammatory cytokines such as IL‐2, ‐4, ‐10, ‐11, and ‐12, remain unchanged when cells senesce.^187^ Also, almost all the stimuli including replicative exhaustion, X‐ray irradiation, chromatin disruption, and telomere shortening that lead to DNA damage can invoke the DDR and produce senescence with the SASP. However, not all cellular senescence is induced by DNA damage: constitutive p38 MAPK activation is also sufficient to induce senescence and SASP.^188^ SASP and p16 expression are correlated, but the occurrence of p16 does not always correspond with other senescence markers such as cell‐cycle arrest.^189^ Furthermore, compared with other pathways of senescence induction, oncogenic RAS‐driven senescent fibroblasts oversecrete ILs, especially IL‐6.^190^ Although the cell cycle arrest occurs within 24 h after suffering damage, senescence always takes several days to become established and various features take different times to occur. For example, p21 is an early marker, but p16 is a late one.^191^

The SASP is dynamically regulated by a variety of nuclear and cytoplasmic factors at numerous levels from chromatin modification, transcription, posttranslational modification, to simple secretion. In addition, SASP can be transmitted to surrounding cells in a noncell autonomous fashion, demonstrating a strong, sensitive global amplification mechanism.^192^ Correspondingly, senescence is easily prevented by interfering with SASP genes because of the positive autocrine and paracrine feedback loops. The generation and regulation of the SASP is influenced by several well‐studied variables, including DNA damage, CCFs, transposable elements, TLRs, and their related regulators. Although different pathways, such as p38MAPK, Janus kinase (JAK)2/STAT3, phosphoinositide‐3‐kinase (PI3K) pathway, mammalian target of rapamycin (mTOR), inflammasomes, and GATA4/p62‐mediated autophagy have all been shown to trigger the SASP, most of them eventually lead to activation of CCAAT/enhancer‐binding protein β (C/EBP‐β) and NF‐κB signaling.^193^ These two transcription factors are more prevalent in the chromatin fractions of senescent cells and work in concert to control the transcription of SASP factors like IL‐1α, IL‐6, and IL‐8, which in turn reinforce the senescent state by positively regulating NF‐κB and CEBPβ activity and enhancing SASP signaling.

### 3.4 Immunosenescence

It should be noted that senescence can occur in many different cell types within the TME, including fibroblasts, immune cells such as macrophages and T lymphocytes, as well as endothelial cells^57^, ^194^ (Table 1). A senescence process occurs in immune cells or even the immune system is termed immunosenescence. In this section, we will outline the types of immune cells that can undergo senescence, describe their characteristics during senescence, and introduce the impact of immunosenescence on tumor progression and antitumor treatment.

**TABLE 1:** Summary of cell types that can undergo senescence.

Cell type	Representative SASP factors	Effects on tumors	References
Fibroblast cell	HGF, MIF, CCL2	Enhanced stemness	^195^
	IL‐6, IL‐8 MMP1, MMP3, MMP13	Chemoradiotherapy tolerance, invasion, metastasis	^81^, ^196^, ^197^, ^198^
	IL‐6, CXCL9, TGFβ	Escape from immune surveillance	^199^
	VEGF	Angiogenesis	^200^
	MMPs, IL‐6	Tumor growth	^201^, ^202^
	IL‐8, CXCL9	EMT, chronic inflammatory microenvironment, tumor growth	^203^, ^204^, ^205^
	CXCR2	Exacerbating cell arrest	^206^
	TGF‐β, CCL2	Tumor suppression	^207^
Mesenchymal stem cells	miR‐146a	Angiogenesis	^208^
	IL‐6	Promoting proliferation and migration	^209^
Liver cancer (hepatocyte, hepatic stellate cell)	EphA2	Promoting cell proliferation	^210^
	PGE2	Suppression of antitumor immunity, promoting tumor growth, drug tolerance, invasion, metastasis	^211^
	Chemokine growth‐regulated alpha protein	Reprogramming the matrix microenvironment to promote tumor growth and metastasis	^212^
	CCL2	NK cells recruitment and MDSC differentiation to remove tumor cells	^213^
	IL‐1α	Immune‐mediated senescent cell clearance, limiting liver cancer development	^214^
	CXCL1, MMP14	Promote tumor invasion	^215^
Breast cancer (epithelial cell)	Eotaxin, CXCL5, Rantes	Promoting tumor growth	^216^
	MMPs	Promoting invasion and metastasis	^201^
Ovarian cancer (epithelial cell)	IL‐6, IL‐8, GM‐CSF, CCL20 mRNA	Inhibiting T cell response to promote tumor growth	^217^, ^218^
	CXCL1, MMP14	Promoting tumor invasion	^215^
Prostate cancer (epithelial cell)	FGF1, growth/differentiation factor 15, insulin‐like growth factor‐binding protein 5	Promoting tumor progression	^219^
	COX2	Promoting tumorigenesis	^171^
	CXCL1 CXCL2	Bone marrow cell recruitment to inhibit tumor growth	^220^
Skin cancer (squamous epithelial cells)	IL‐6, MMP3, MMP9, CXCL10	Driving tumorigenesis	^218^
Melanoma (melanocyte)	CCL5	Lymphocyte recruitment to inhibit tumor growth	^221^
Thyroid cancer (thyroid follicular cell)	CXCL12	Anoikis resistance	^222^
T cell	Galectin 9/TIM3	Antitumor	^223^
Macrophage	IL‐6, IFN‐γ	M1 polarizing to inhibit tumor growth	^224^, ^225^

Abbreviations: EMT, epithelial interstitial transformation; MMP, matrix metalloproteinases.; VEGF, vascular endothelial growth factor.

Many factors that elicit SASP, such as cGAS–STING pathway, NF‐κB, and C/EBPβ can induce senescence in immune cells and affect their function.^226^, ^227^, ^228^ In addition, the recruitment of p38 to the scaffold protein TAB1 in T cells driven by low‐nutrient levels or DNA‐damage signaling can induce the senescence of human T cells by inhibiting telomerase activity and T cell proliferation.^229^ Various immune cell subsets are changed during immunosenescence. The number of naive T and B cells is decreased, while memory T and B cells proliferate and display senescence‐related traits. Senescent T cells exhibit abnormal phenotypes, including downregulation of costimulatory molecules such as CD27 and CD28, and upregulation of killer cell lectin‐like receptor subfamily G, CD57, Tim‐3, and cytotoxic T‐lymphocyte‐associated protein 4, which are closely related to malignant tumors.^16^ Moreover, the amount of the two cell cycle regulators, p16 and p21, was upregulated along with the decrease in CD27 and CD28. The diversity of T‐cell receptors (TCRs) decreases with age in the aging process, and the number of mature NK cells is also affected, as evidenced by the reduced output of immature CD56^bright^ cells and the accumulation of highly differentiated CD56^dim^ NK cells.^230^ In addition, the level of NK cell activation markers, including DNAM‐1, NKP30, and NKP46, is decreased, while inhibitory receptors, such as KIR and NKG2C, are increased in the process of immunosenescence.^231^ The proportion and quantity of CD19^+^ B cells in peripheral blood are decreased, reflecting a change in the distribution of mature B cells,^232^ and the function of B cells is altered by a decrease in the autoimmune regulator AIRE and the autoantigen gene in thymic B cells. Alterations also occurred in other players of the immune system (Table 2). The main organizers of the immune response, dendritic cells, exhibited diminished abilities for antigen presentation, endocytosis, and IFN production,^233^ and neutrophils lost their capacity to phagocytose pathogens during immunosenescence.^234^ Although it is unclear if aging affects the phagocytosis and antigen presentation ability of macrophages, a number of diseases, including cancer, can emerge as a result of immunosuppressive disorders and chronic low‐grade inflammation caused by age‐associated tissue‐specific macrophages and neutrophils.^235^ Myeloid‐derived suppressor cells (MDSCs) build up in the bone marrow, blood, and spleen of tumor‐bearing old mice and cause immunosuppression that hinders the removal of senescent cells and tumor cells.^236^ In addition to promoting immunosenescence, the expansion of MDSCs may also generate detrimental age‐related bystander effects in host tissues by secreting TGF‐β and IL‐10, which might lead to disorganization of the ECM and cause tumor metastasis in elderly patients.^237^, ^238^ Therefore, alterations in immune cell subsets during senescence are intrinsically linked to tumor progression and their inclusion as subjects of cancer research is important.

**TABLE 2:** Changes in various immune cell subsets during immunosenescence.

Immune cell type	Markers	Expression level up (↑) or down (↓)	References
T cell	CD27, CD28	↓	^239^, ^240^
	CD57, KLRG‐1, Tim‐3, TIGIT, CD45RA	↑	^239^, ^240^
	P16, P21, P53	↓	^1^, ^240^, ^241^
	IL‐7	↓	^242^
	IL‐10, TGF‐β	↑	^243^, ^244^
	SAHF, ZAP70, MRE11A	↓	^245^, ^246^
	P21, P16	↑	^246^
	Glycolysis	↑	^247^, ^248^
	Mitochondrial biogenesis	↓	^247^, ^248^
	ROS, p38 MAPK, GDF15	↑	^229^, ^247^, ^248^
	IFN‐γ, TNF‐α, IL‐65 and GzmB	↓	^249^
	Perforin, GzmB	↓	^243^
	SHP‐1	↑	^250^
	DUSP6, CD3	↑	^251^
	Lck, ZAP70, DLG1, Lat, SLP‐76	↓	^243^, ^252^
	IL‐6, IL‐8, IFN‐γ, TNF,	↑	^109^, ^253^, ^254^, ^255^
	Telomere	↑	^65^, ^256^
	SIRT1, AMPK, NDA	↓	^257^
	IGF1R	↑	^258^
	TPPII	↓	^259^
	TGFβR3, INF‐β, IL‐6, IL‐27	↑	^260^, ^261^
NK cell	SIRT1, AMPK, NDA	↓	^257^
	Telomere	↑	^65^, ^256^
	p16	No change	^262^
	NKp30, NKp46, DNAM‐1, NKG2A, NCR	↓	^231^, ^262^, ^263^, ^264^
	KIR, NKG2C, CD57, CD94	↑	^231^, ^263^, ^264^, ^265^
Monocytes/macrophages	CD62L, TLR1/4	↓	^264^
	CD11b, TLR5	↑	^264^
	IL‐5	↑	^266^
	IFN‐α, IL‐1β, TNF‐α, IL‐6, IFN‐γ	↑	^267^
	TLR	↓	^268^
	CCL2, CCL7	↓	^269^
B cell	P16	↓	^270^
	AID	↓	^271^
	Telomere	↓	^270^
	Atg5, Atg7	↓	^272^, ^273^
Mast cell	FcγRIIB/III	↓	^274^
Myeloid dendritic cell	TNF‐α, IL‐6, IL‐12, IFN‐α, IFN‐β, IFN‐γ	↓	^275^
Dendritic cell	IFN‐α	↓	^276^
	ROS	↑	^276^
Neutrophils cell	ICAM‐1	↓	^277^, ^278^
	CXCR2	↑	
	CD11a, CD11b	No change	^264^

Regarding the essential role of the immune system during tumor development and malignant progression, immunosenescence could affect multiple tumoral processes, which might be one of the most significant reasons why the incidence of most cancers rises with age.^279^ For instance, research suggests that the pathophysiology and management of individuals with breast cancer are profoundly influenced by immunosenescence, particularly CD8^+^ T‐cell senescence.^280^ Reduced IFN signaling in CD8^+^ T cells due to immunosenescence was identified in older mice with breast cancer.^281^ Immunosenescence may impair the response to cancer immunotherapy, as the lack of naive T cells means that new antigens may not be recognized. Despite some ambiguous results, the aging immune system seems to impair the effectiveness of immune checkpoint blockers (ICBs), as evidenced by the fact that elderly patients have shorter progression‐free survival and overall survival (OS) than younger patients,^282^ while in patients with late‐stage melanoma who received ipilimumab treatment, noteworthy baseline frequencies of late stage‐differentiated effector memory CD8 cells (>23.8%) were inversely linked with OS.^283^ These results suggested that a pool of young T cells is necessary during ICB treatment, which is in accordance with the recent observation in a mouse model suggesting that aged or senescent immune cells can accelerate aging and decrease lifespan, whereas young immune cells suppress senescence.^279^ However, further investigations are needed to confirm this conclusion because of the few elderly cancer patients participating in the current clinical trials.

Bispecific T‐cell engagers are recombinant proteins made of two single‐chain variable fragments from two different antibodies that simultaneously target tumor‐specific antigen and effector T‐cells (mostly CD3) for activating T cells that are adjacent to tumor cells.^284^ Accordingly, the classical features of lymphocyte senescence–loss of CD28 expression on T cells, will limit their antitumor efficacy. Recent data have demonstrated that the CD28‐mediated expression of CD80 or CD86 on cancer cells can increase the cytotoxicity of blinatumomab, a bispecific T‐cell engager targeting CD19 and CD3 to treat Philadelphia chromosome‐negative B‐cell acute lymphoblastic leukemia, as does cotreatment with a monoclonal CD28 antibody.^285^ When combined with anti‐PD‐1 antibodies, the use of bispecific antibodies targeting PSMAxCD28 and EGFRxCD28 has demonstrated strong antitumor activity.^286^ The effect of chimeric antigen receptor T (CAR‐T) cell therapy, another immunotherapeutic that induces T cells to express tumor‐specific antigen receptors, is also highly dependent on functionally active T cells to recognize and eliminate tumor cells in an HLA‐independent manner.^287^ Because the reconstructed and expanded CAR‐T cells were exposed to a harsh TME, a high frequency of senescent cells was observed in the generated CAR‐T products. As reported by Zhu et al.,^288^ the T‐cell senescence marker CD57 can easily and efficiently transfer from glioblastoma stem cells to CAR‐T cells and cause their senescence. CD57 can also be elicited to induce CAR‐T‐cell senescence when CD133‐specific CAR‐T cells destroy patient‐derived glioblastoma stem cells.^288^ Furthermore, when CAR is introduced into T cells with distinct TCRs, the presence of TCR antigen results in the loss of CD8^+^ CAR‐T‐cell potency, which is linked to T‐cell senescence, exhaustion, and apoptosis.^289^ In general, improving the anticancer impact of modified T cells requires reactivating senescent CAR‐T cells. To test the idea that immunosenescence affects the effectiveness of immunotherapy, a focused investigation of immunosenescence indicators in upcoming clinical studies is required. Additional investigations into specific immunoregulatory pathways should aid in identifying new, more precise immunosenescence targets as well as novel treatment approaches.

## 4 EXPLOITING SENESCENCE FOR CANCER THERAPY

Given the significant role of senescence in tumors, promising strategies are being developed to enhance its tumor‐suppression effect and eliminate or reduce the senescence‐related adverse consequences. The one‐two punch therapy designed to induce senescence of tumor cells and subsequently to clear out senescent cells or block their negative side effects has attracted great interest among researchers and the clinical community. Punch 1 could involve immunotherapy, molecularly targeted medications, and spatially targeted radiation to induce senescence in the tumor, stroma, and bystander tissue. Punch 2 would be used if required to mitigate any adverse effects from the drugs or the senescent cells to prevent tumor recurrence, drug resistance, plasticity, or normal tissue injury.

**FIGURE 4:**
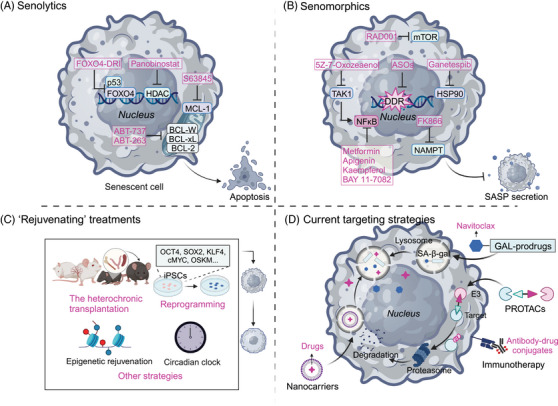
Current strategies and technologies for rationally targeting cellular senescence for cancer therapy. Senotherapy, including senolytics, senomorphics, and senorevertors that selectively target senescent cells, has been explored for effective tumor treatment. Robust targeting strategies such as Gal‐prodrugs, PROTACs, nanocarriers and immunotherapy will improve their efficiency. (A) One method is to use antisenescence chemicals known as senolytics to selectively remove/clear senescent cells. (B) The other strategy involves blocking induction of the SASP by drugs called senotherapeutics. (C)The recently reported rejuvenation approach hold promise of a third choice for targeting senescent cells. (D) the development of senotherapeutics that combine transdisciplinary technologies and strategies that facilitate targeting to senescent cancer cells are rapidly gaining interest. PROTACs, proteolysis‐targeting chimeras; SASP, senescence‐associated secretory phenotype.

“Senotherapeutics” generally include three categories: “senolytics,” which are targeted to remove senescent cells; “senomorphics,” which regulate the activity of senescent cells, including SASP, rather than eliminating them; and most recently, “rejuvenating” treatments, which reverse senescence and convert the cells from the senescent state to the growth state, wherein they enter the cell cycle.^290^ Currently, the combined use of senotherapeutics and classical anticancer therapies has been confirmed as effective for cancer treatment since this combination strategy can effectively decrease the negative effects of cancer treatments, and minimize the chance of chemotherapy resistance and cancer recurrence.^22^, ^291^ In this section, we describe promising therapeutic regimens that promote the positive and avoid the negative effects of cellular senescence for cancer management and provide an appraisal of their efficacy and safety based on current clinical trials. The emerging technologies and strategies that have been employed in developing senotherapeutics will be introduced (Figure 4).

### 4.1 Categories of “senotherapeutics”

#### 4.1.1 Senolytics

Persistent senescent tumor cells frequently upregulate the expression of apoptosis inhibitors and remain metabolically active, promoting proliferation and metastatic dissemination.^292^ These include antiapoptotic pathway (SCAP) factors such as the BCL‐2 family (including BCL‐2, BCL‐XL, and BCL‐W), and the prosurvival pathways such as PI3K/Akt, tyrosine kinases, HSP90, and HIF‐1α.^293^, ^294^, ^295^ Senolytics have emerged as a promising therapeutic strategy to selectively eliminate senescent cells by interfering with their survival pathways.

The HDAC inhibitor, panobinostat, was demonstrated to induce chromatin relaxation and boosts DDR signaling with consequent apoptotic cell death.^296^ It has been reported as a successful senolytic for effectively killing persistent senescent cells that occur in non‐small cell lung cancer (NSCLC) and head and neck squamous cell carcinomas (HNSCC) following cisplatin or taxol treatment.^297^ Other well‐demonstrated senolytics being tested for cancer treatment are inhibitors of BCL‐2 family members. For example, ABT‐737 and ABT‐263 were shown to selectively kill senescent cells and blunt the recurrent growth and aggressiveness of cancer cells via interference with BCL‐2, BCL‐XL, and BCL‐W.^291^, ^298^, ^299^ Other BCL‐XL inhibitors, such as A‐1331852 and A‐1155463, have also exhibited senolytic activity,^300^, ^301^ even though their efficacy in tumor therapy has not been confirmed. Using single‐cell RNA sequencing, Martina Troiani and her colleagues identified novel senolytic targets that were essential for the survival of senescent tumor cells. Their results suggested a potential target for cancer therapy by inhibition of Mcl‐1 using S63845 to eliminate senescent tumor cells and thus prevent their metastatic dissemination.^302^ In a radiotherapy‐treated NSCLC model, specific induction of apoptosis in senescence‐like cancer‐associated fibroblasts using FOXO4‐DRI (a FOXO4‐p53‐interfering peptide) led to the significant radiosensitivity of NSCLC cells both in vitro and in vivo.^303^ A number of organic substances, including quercetin and fisetin, either alone or in combination with chemotherapeutic drugs like dasatinib, a pan‐tyrosine kinase inhibitor, exhibited senolytic effects in a variety of contexts in vitro and in vivo^304^, ^305^ and have been reported to enhance the efficacy of chemotherapy and suppress tumor progression.^306^, ^307^ Taken together, these studies demonstrated that senolytics provide a promising strategy for improving the efficacy of cancer therapeutics by interfering with antiapoptosis‐associated pathways and selectively eliminating senescent cells.

#### 4.1.2 Senomorphics

Senomorphics are used to disrupt key attributes of senescent cells while keeping the cells alive. For example, this approach could interfere with pathways mediated by p38 MAPK, NF‐κB or mTOR to suppress SASP expression and delay key aspects of aging and aging‐associated diseases. Although rapamycin, an inhibitor of mTOR, also works through senescence‐independent mechanisms, treatment with rapamycin (or its analog RAD001) was reported to block the protumorigenic SASP by selectively interfering with the translation of IL1α.^308^ Metformin, an antidiabetic drug, has been proven to efficiently suppress the secretion of proinflammatory SASP factors by preventing the signaling events that are required for full‐spectrum SASP expression, including the nuclear translocation of NF‐κB, the phosphorylation of IκB and IKKα/β, and the induction of autophagic flux,^309^, ^310^, ^311^ therefore offering a novel, targeted therapeutic strategy for cancers.^312^ Other NF‐κB signaling modulators, including apigenin, kaempferol, and BAY 11−7082, have also been demonstrated to reduce the SASP and exhibit tumor‐suppressive effects.^313^, ^314^ Inhibition of HSP90 blunted the release of several cytokines in chemotherapeutically‐treated malignant pleural mesothelioma cells, which resulted in the suppression of FAK–AKT signaling and the inhibition of 3D growth and migration.^315^ Similarly, inhibitors targeting the activator of transcription (STAT), JAK/signal transducer, IL‐1, IL‐6, and tumor necrosis factor (TNF) are effective, alone or in synergy with senescence‐inducing chemotherapy, to effectively abrogate the proliferation and migration of cancer cells.^316^, ^317^, ^318^, ^319^

Pharmacologically targeting TAK1, the serine/threonine protein kinase that is functionally implicated in the regulation of SASP development, should reduce the SASP and prevent tumor cells from acquiring resistance factors from damaged stromal cells in the TME, resulting in significant tumor regression.^320^ Likewise, inhibition of nicotinamide phosphoribosyltransferase (NAMPT) can suppress the outgrowth of cisplatin‐treated epithelial ovarian cancer (EOC) via inhibition of senescence‐associated cancer stem cells (CSCs) because NAD^+^/NADH metabolism is a crucial controllable factor in determining the magnitude of the proinflammatory SASP.^321^ Combined utilization of the NAMPT inhibitor FK866 and the chemotherapy drug, cisplatin, improved the survival of EOC‐bearing mice, representing a promising therapeutic strategy by suppressing TIS‐associated CSCs.^322^ Last, inhibition of telomeric DDR was explored to reduce senescence during cancer treatment. The use of antisense oligonucleotides that specifically target telomere, transcription‐related regulatory factors and damage repair regulators were observed to effectively reduce DDR activation and the expression of senescence markers in various diseases, including Hutchinson‐Gilford progeria syndrome and castration‐resistant prostate cancer.^323^, ^324^, ^325^ Altogether, senomorphics or similar agents that do not eliminate senescent cells but rather inhibit their attributes may offer a viable alternative to senolysis.

#### 4.1.3 Rejuvenating” treatments

Recent studies suggest that certain cell types such as embryonic senescent cells^326^ and dermal fibroblasts^327^ can be reverted to reenter the cell cycle, although senescence has long been considered an irreversible cell fate. Rejuvenation, which previously was deemed an unrealistic panacea for age‐related diseases, has recently been reintroduced as a way to reverse rather than just attenuate senescence with verified alteration of aging biomarkers and physiological readouts.^328^ Three categories of rejuvenation interventions have been reported to achieve a robust, sustained, and systemic decrease in biological age: (1) parabiosis and transplantation, (2) reprogramming, and (3) other strategies.

Parabiosis, a surgical procedure that connects young and old animals by creating a linked circulation, has been shown to improve the performance of aged organs.^329^ For example, the declining proliferation of hepatic progenitor cells in old mice was restored by exposure to a young systemic environment through activation of Notch signaling as reported by Conboy et al.^330^ This intermittent blood and plasma exchange can lead to a significant reduction in cellular senescence in multiple types of aged tissues exposed to young blood or plasma,^331^ while young animals exposed to aged blood or plasma exhibited higher levels of senescence markers.^332^, ^333^ Expanding upon these studies, some researchers have also proposed transplanting organs such as ovaries or cells such as bone marrow and splenocytes to ameliorate age‐related problems, improve overall health, and extend the healthy lifespan.^334^ A recent study found that transplanting young immune cells into old mice decreased senescence, whereas the transplantation of old immune cells promoted senescence.^279^

Another noteworthy intervention is a reprogramming strategy that employs a selected set of factors to convert differentiated somatic cells into a specific desired cell type. For example, the methods of promoting the expression of Yamanaka factors (OCT4, SOX2, KLF4, and cMYC) for inducing pluripotency have achieved increasing success.^335^, ^336^ The induced pluripotent stem cells are able to transform aged cells into more youthful versions by mechanisms including lengthening telomeres, alleviating oxidative stress, reorganizing the mitochondrial network, and so on.^337^ Several other reprogramming techniques such as lineage reprogramming and partial reprogramming are being developed to provide insights into new techniques of regenerative medicine for aged patients.^328^ Other, even more ambitious rejuvenation strategies are emerging: epigenomic remodeling employs biochemical modifications of genes to alter the regulation of gene transcription in response to physiological stimuli.^338^, ^339^, ^340^ These strategies are all based on rejuvenating treatments as a third therapeutic option to reverse senescence and, thus, they should be investigated as an effective adjunct to cancer treatments.

Among these three kinds of senotherapeutics, senolytics offer the greatest advantage because continuous administration of SASP inhibitors is not required. The senescent cells are eradicated rather than only a single senescence phenotype, and since intermittent administration will suffice, the problem of adverse side effects is reduced.^67^, ^341^ However, it is not known whether prolonged or repeated treatments could eventually become deleterious to an organism. Moreover, it remains to be investigated whether these treatments are detrimental or beneficial in advanced age when the senescent cell burden is relatively high. Such, “rejuvenating” treatments must face even larger challenges due to ethical issues and technical bottlenecks. As promising as it sounds, whether systemic rejuvenation can be achieved and how it can be translated to human applications has a long way to go despite the successes obtained with animal models.

### 4.2 Approaches to discover and develop novel senotherapeutics for cancer therapy

To date, hypothesis‐driven and mechanism‐based drug discovery paradigms assisted by bioinformatics approaches and/or library screening are the most used strategies for the discovery of new senotherapeutics.^25^ With an increased understanding of the features of senescence and the recognition of the great translational potential of senotherapeutics, novel approaches are being developed to promote the discovery and development of rational, personalized treatments. Senoprobes, molecules specifically designed to detect, identify, and quantify senescent cells, have garnered much clinical interest because of their potential use in tracking cellular senescence in a variety of age‐associated diseases.^342^ According to the observed alteration of various senoprobes, such as SA‐gal‐based chemogenic or fluorogenic dyes, cell‐based chemical screening, and genetic screening of vulnerabilities of senescent cells have been established for drug development.^343^, ^344^ To supplement senotherapeutic screening with a deeper understanding of the biology of senescence and the identification of senescence targets, additional technologies have been developed, including structure‐based virtual screening, computer‐aided drug design (CADD), artificial intelligence (AI), and machine learning (ML). Rational drug design directed by CADD, AI, and ML replenishes our libraries with new chemical entities by the discovery of senotherapeutics with unique chemical structures. Furthermore, by conducting numerous rounds of structure–activity relationship studies, drugs can be designed to order for achieving desired qualities, like bioavailability and cell targeting. By combining drug screening and design strategies, a plethora of novel potential senotherapeutics can be identified to eradicate senescent cells, and which could also come into play in cancer drug treatment strategies.

### 4.3 Strategies for effectively targeting senescent cells in cancer therapy

Growing interest is being paid to developing novel senotherapeutics by incorporating multidisciplinary technologies such as biology, chemistry, immunology, and nanotechnology, for more effective cancer therapy. In addition to conventional senotherapeutics and their combination use, current senotherapeutics mainly include prodrugs, protein degrading systems, nanocarriers, and immunotherapies.

#### 4.3.1 Prodrugs

By covalently joining galactose or acetyl galactose groups to a cytotoxic molecule, enhanced lysosomal SA‐β‐gal activity was used to construct galactose‐based prodrugs. The cytotoxic moieties are generally chemotherapeutic reagents and can be released in senescent cells to selectively kill them. For instance, Nav‐Gal, the galacto‐conjugant of Navitoclax, was reported to enhance the cytotoxicity of cisplatin in human A549 lung cancer cells, and concomitant treatment with cisplatin and Nav‐Gal eradicated senescent lung cancer cells and significantly reduced tumor growth with reduced Navitoclax‐induced platelet apoptosis.^345^ Similar results were obtained in another study of triple‐negative breast cancer (TNBC), in which the combination of palbociclib with Nav‐Gal delayed tumor growth and reduced metastases in an aggressive human TNBC‐derived mouse xenograft model through successful senescence induction and the subsequent removal of senescent cells.^346^ These studies offer an efficient strategy of combining senescence induction with senolysis for cancer treatment and provide a targeted approach to develop effective and safer therapeutics.

#### 4.3.2 Protein degraders

Proteolysis‐targeting chimeras (PROTACs) are being developed to induce the degradation of a protein of interest (POI) by employing the ubiquitin‒proteasome system. It is composed of a ligand such as a small‐molecule inhibitor that recruits the POI, a covalently linked ligand for recruiting E3 ligase, and a malleable bridge connecting the two ligands. To date, many PROTAC‐targeted proteins that were known drug targets have been successfully exploited for cancer therapy,^347^ including several PROTAC‐based senolytics. An example is ARV825, a ternary complex including the BET inhibitor OTX015 for degradation of BET family proteins.^348^ ARV825 demonstrated strong senolytic action and accelerated BRD4 degradation by attenuating NHEJ and upregulating autophagic gene expression, thereby eliminating senescent cells and reducing the development of several types of tumors, such as thyroid carcinoma,^349^ TNBC,^350^ and glioma.^351^ However, PROTACs may have less optimal pharmacokinetic properties because of their higher molecular weight.^352^ Before further SCAP targets can be used to create PROTAC senotherapeutics and use them to treat cancer patients, much additional research is required.

#### 4.3.3 Nanotechnology

Nanotechnology engineers design and manufacture materials at the atomic and molecular scale to allow controlled delivery and release of various payloads. Nanomethods have been applied to treat human disease in almost every field, including cancer, by enabling detection, diagnosis, and drug delivery. Nanocarriers targeting senescent cells for detection and therapeutic intervention have been developed using a wide variety of nanomaterials, particularly in the form of nanoparticles (NPs), and several of these exhibited potent antitumor activity by acting as senotherapeutics. By creating NPs conjugated with galacto‐oligosaccharides, the well‐characterized SA‐β‐gal can be utilized for the preferential delivery of drug payloads into senescent cells.^353^ Self‐assembling peptides can be modified with galactose groups to trigger senescent cell death by forming intracellular nanofibers and hydrogels and then activating apoptotic pathways after being selectively taken up by senescent cells and broken down by SA‐β‐gal, indicating the potential of supramolecular nanomaterials for the detection and treatment of cancer.^354^ Employing senescence‐associated surface proteins, such as β_2_‐microglobulin (B_2_M), provides another strategy for the precise delivery of NPs to senescent cells through generation of molecularly imprinted nanopolymers (nanoMIPs).^355^

Whether NPs synthesized from other kinds of materials including zinc oxide, molybdenum disulfide, or Fe_3_O_4_, can be designed to preferentially target senescent cells and deliver anticancer treatments remains largely unexplored.

#### 4.3.4 Immunotherapy

An alternative senotherapeutic approach that boosts the immune system's capacity to eliminate senescent cells involves immunotherapy. It commonly utilizes senescence‐specific proteins that are particularly increased on the surface membranes of senescent cells, such as B2M, urokinase‐type plasminogen activator receptor (uPAR), dipeptidyl peptidase 4 (DPP4), glycoprotein nonmetastatic melanoma protein B (GPNMB), and so on. Senescent cells can recruit immune cells by secreting proinflammatory cytokines and chemokines and eventually undergo immune‐mediated clearance. For example, in pancreatic ductal adenocarcinoma with a mutant KRAS, the combination of MEK and CDK4/6 inhibitors triggered a SASP to enhance tumor vascularization, which stimulated the local accumulation of CD8^+^ T cells and made the cells susceptible to PD‐1 checkpoint inhibition.^20^

Antibody‒drug conjugates (ADCs) are an innovative strategy that have been utilized successfully to treat cancers for decades. This technique exploits the specificity of a monoclonal antibody (mAb) to bind to an extracellular epitope for the delivery of attached cytotoxic drugs. For example, senescent cells have been reported to be eliminated by a B_2_M‐based IgG1 monoclonal antibody conjugated to duocarmycin ADC that released duocarmycin following internalization via endolysosomal trafficking and cleavage by cathepsin B.^356^ Several other types of immunotherapies, such as antibody‐dependent cellular cytotoxicity,^357^ senolytic vaccination,^358^ and neutralizing antibodies,^312^ can target senescence‐related surface proteins as well, and these strategies show great promise for improving the efficacy of cancer treatments. In a recent study, CAR T cells were redirected to ablate senescent cells by recognizing the senescent surface protein, uPAR.^359^ In that study, mice with lung cancer treated with a combination medication that induced senescence survived longer when treated with uPAR‐specific CAR T cells. Similarly, CAR NK cells or CAR macrophages could be created to target senescent cells and increase the cytotoxic effect of anticancer therapy.^360^

Only a small percentage of senotherapeutics makes it through clinical trials, despite a wealth of data demonstrating their capacity to sensitize tumors to radiation and chemotherapy in preclinical models. Dasatinib, quercetin, ABT‐263, and fisetin are a few examples (Table 3). Although several clinical trials are underway), more research is required to determine the most effective dosing regimens, particularly with regard to the one‐time versus recurrent administration of senotherapeutics, and to show whether the long‐term advantages of these drugs outweigh any potential toxicities. Notably, prospective findings from large‐cohort cancer trials are urgently needed.

**TABLE 3:** Examples for senotherapeutic and their application in clinical trials.

Senotherapeutics	Evidence	Trials registration number
Navitoclax (ABT‐263)	ABT‐263 could shift senescence response to therapy toward apoptosis by interfering with the interaction between BCL‐XL and BAX.^298^ Senolytic navitoclax (ABT‐263) selectively eliminate senescent PCa cells following ADT and interfered with the development of androgen‐independent outgrowth.^361^ PARPi‐induced senescence renders ovarian and breast cancer cells transiently susceptible to senolytic drugs.^362^	NCT00445198 NCT00481091 NCT00445198 NCT01087151 NCT00788684 NCT02591095 NCT00406809
ABT‐737	ABT‐737, navitoclax, chloroquine, ATMi, ATRi, BV‐6, PX‐866 and the natural compounds fisetin and artesunate exhibit senolytic activity, inducing death in senescent cells more efficiently than in proliferating cells.^299^ ABC294640 acted synergistically with ABT‐737 (a Bcl‐2 inhibitor) in inducing myeloma cell death.^363^ Senescent PanIN cells exert a tumor‐promoting effect, Bcl2‐family inhibitor ABT‐737 eliminated Cox2‐expressing senescent cell.^364^	NCT01440504
Sertraline	Antidepressant sertraline as an agent that kills hepatocellular carcinoma cells that have been rendered senescent by inhibition of CDC7.^365^	NCT02891278 NCT02770378
Cardiac Glycosides)	Senescent cells present a slightly depolarized plasma membrane and higher concentrations of H^+^, making them more susceptible to the action of CGs.^366^	NCT00650910 NCT03928210
Panobinostat	Panobinostat kills persistent senescent cells that accumulate during standard chemotherapy in NSCLC and HNSCC.^297^	NCT02717455
Fisetin	Fisetin can reverse chemotherapy resistance that was elicited by eIF3A R803K somatic mutation induced senescence.^367^	NCT04733534
Venetoclax (ABT‐199)	Venetoclax reduced doxorubicin‐induced senescent cell subpopulations and significantly enhanced the apoptotic effect of doxorubicin.^368^	NCT05893472

Abbreviations: ADT, androgen deprivation therapies; CGs, cardiac glycosides; HNSCC, head and neck squamous cell cancer; NSCLC, non‐small cell lung cancer.

*Data sources*: PubMed; clinical registration website.

## 5 SUMMARY AND PERSPECTIVES

**FIGURE 5:**
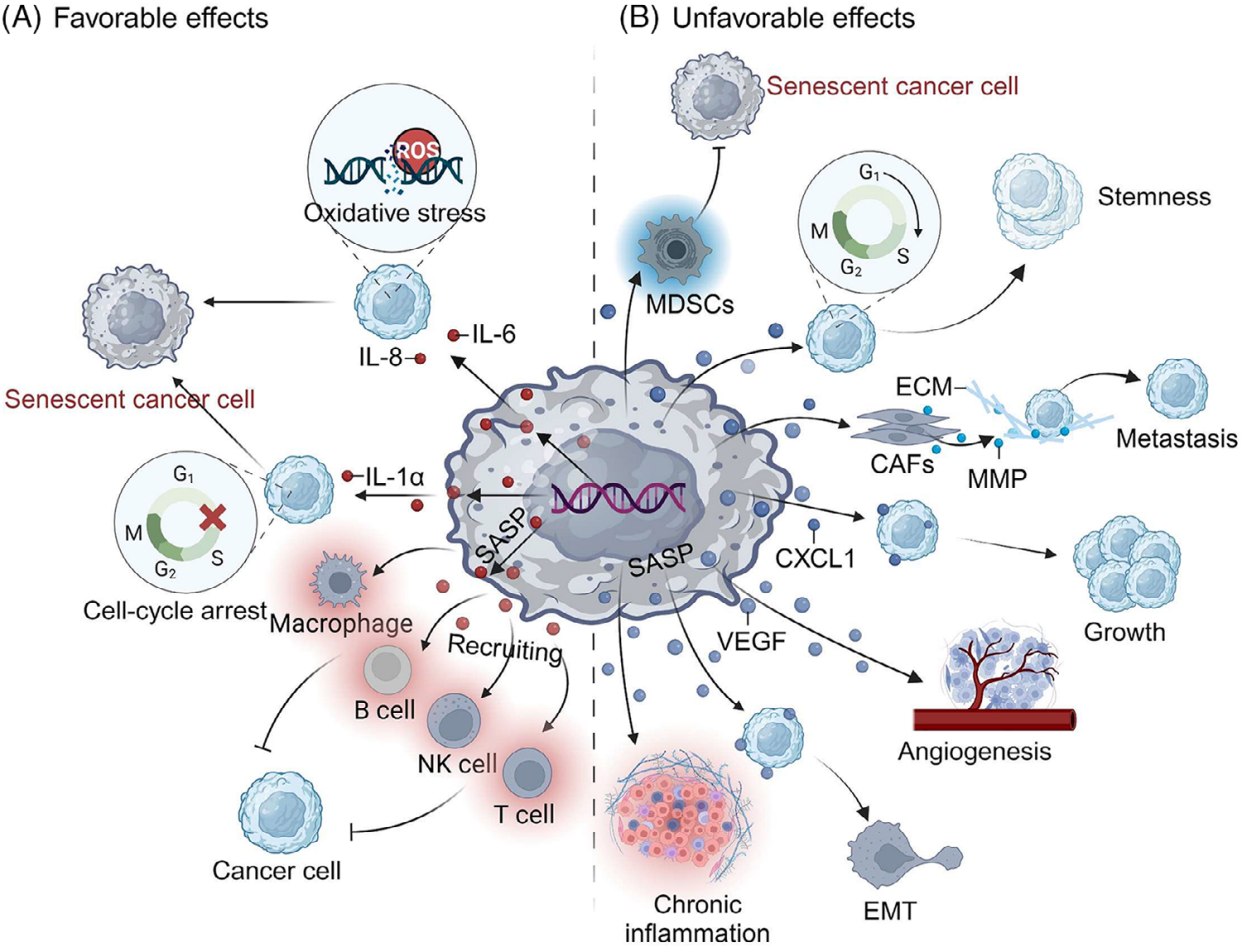
Senescence exerts double‐edged sword effects on tumor biology. (A) Senescence may create a barrier for tumorigenesis and act as a tumor suppressor upon treatment, (B) while activation of the SASP may promote tumor progression in some contexts. MDSCs, myeloid‐derived suppressor cells; ECM, extracellular matrix; CAFs, cancer‐associated fibroblasts; MMP, matrix metalloproteinases; EMT, epithelial interstitial transformation; VEGF, vascular endothelial growth factor.

Senescence has been clearly demonstrated to participate in multiple aspects of the pathogenesis and treatment outcomes of cancer. Senescence frequently occurs in tumor cells, especially when exposed to chemotherapy or radiation. These senescent tumor cells secrete multiple cytokines and chemokines to dynamically modulate the surrounding tissue microenvironment and the immune response, such as by vascular reconstruction, immune escape, avoidance of apoptosis, maintenance of tumor dormancy, and promotion of invasion and metastasis. Virtually all the other cell types in the TME can undergo senescence, which inevitably affects the malignant features of tumor cells. The activation of SASP and secretion of associated proteins is the most effective mechanism by which senescence expands into the microenvironment and alters tumor phenotype. However, signals from senescent cells may inhibit tumor progression through immune surveillance functions and maintaining homeostasis. In some contexts, immune factors and secretory factors can recruit immune cells and even improve the vascular penetration of drugs (Figure 5). Therefore, exploiting the tumor‐inhibitory effects of cellular senescence and avoiding its deleterious effects should help to rebuild appropriate tissue and cell signals, thereby facilitating tumor management.

Three categories of senotherapy including the use of senolytics, senomorphics, and recently emerging rejuvenation strategies have been widely tested and shown to be effective in eliminating senescent cells or altering their phenotypes. Dasatinib and quercetin are two of these senotherapeutics that have achieved promising results from preclinical studies and have facilitated progression to early‐phase clinical trials. In addition, multidisciplinary technologies such as the use of senoprobes, nanotechnology, prodrugs, protein degraders, and immunotherapy are being successfully employed to complement conventional senotherapeutics. However, there is still a long way to go before the results from experimental models can be fully translated into the clinic.

From early cell cycle arrest to the later stages of dormancy, senescence is a dynamic and heterogeneous process that engages complex molecular pathways.^369^ In addition, senescent phenotypes vary among different cell types and exhibit differences depending on the triggers. For example, not all SASP components are simultaneously activated, and p16‐induced senescence does not always activate the SASP transcriptional program. The fact that no single marker can adequately reflect senescent cells represents a significant constraint on the study of senescence. A panel of markers, when present together, are currently used to identify senescent cells. As such, it will be crucial to first conduct transcriptomic and proteomic analyses at the single‐cell level in pertinent cell and tissue types to identify the most widespread indicators of the senescent state to simplify identification of senescent cells.

Next, more convincing and robust in vivo models for studies of senescence in tumor cells need to be established. Despite the well‐demonstrated association between senescence and tumor progression and its management, more investigation is still required to identify the determinants of cellular senescence and the molecular mechanisms connecting senescence and malignant events for optimal, personalized tumor treatment. Last, despite the enormous efforts put into developing “senotherapeutics,” the majority of them are still in the early stages of development, rarely in clinical trials, let alone used for oncotherapy in clinical settings. An extensive portion of our knowledge regarding the role of senescent cells in illness is derived from animal models of human ailments. Senolysis in humans has not yet been proven to be safe or effective, which is a requirement before developing patient treatments. A better understanding of the underlying mechanism of senescence and its molecular connections with malignant tumors is an essential requirement for the clinical application of these new findings to actual cancer patients. Through a combination of advanced technical and drug delivery approaches, an expanded range of biomarkers, and 3D in vivo models of aging and tumors, we are confident that modulating cellular senescence via the application of senotherapeutics will take its place as a gold standard for cancer treatments.

## AUTHOR CONTRIBUTIONS

X. K. and Z. C. G. conceived the structure of the manuscript. J. P. and D. X. R. drafted the initial manuscript. X. K. and Z. C. G. revised the manuscript. L. L. and Z. P. prepared the figures. All authors read and approved the final manuscript. J. P., D. X. R., and L. L. contributed equally to this work.

## CONFLICT OF INTEREST STATEMENT

The authors declare no potential conflict of interest.

## ETHICS STATEMENT

Not applicable.

## Data Availability

Not applicable.
